# Analysis and visualization of the effect of multiple sclerosis on biological brain age

**DOI:** 10.3389/fneur.2024.1423485

**Published:** 2024-10-10

**Authors:** Catharina J. A. Romme, Emma A. M. Stanley, Pauline Mouches, Matthias Wilms, G. Bruce Pike, Luanne M. Metz, Nils D. Forkert

**Affiliations:** ^1^Department of Radiology, Cumming School of Medicine, University of Calgary, Calgary, AB, Canada; ^2^Department of Pediatrics, Cumming School of Medicine, University of Calgary, Calgary, AB, Canada; ^3^Hotchkiss Brain Institute, Cumming School of Medicine, University of Calgary, Calgary, AB, Canada; ^4^Alberta Children's Hospital Research Institute, Cumming School of Medicine, University of Calgary, Calgary, AB, Canada; ^5^Department of Clinical Neurosciences, Cumming School of Medicine, University of Calgary, Calgary, AB, Canada

**Keywords:** brain age prediction, deep learning, explainable artificial intelligence, magnetic resonance imaging, multiple sclerosis

## Abstract

**Introduction:**

The rate of neurodegeneration in multiple sclerosis (MS) is an important biomarker for disease progression but can be challenging to quantify. The brain age gap, which quantifies the difference between a patient's chronological and their estimated biological brain age, might be a valuable biomarker of neurodegeneration in patients with MS. Thus, the aim of this study was to investigate the value of an image-based prediction of the brain age gap using a deep learning model and compare brain age gap values between healthy individuals and patients with MS.

**Methods:**

A multi-center dataset consisting of 5,294 T1-weighted magnetic resonance images of the brain from healthy individuals aged between 19 and 89 years was used to train a convolutional neural network (CNN) for biological brain age prediction. The trained model was then used to calculate the brain age gap in 195 patients with relapsing remitting MS (20–60 years). Additionally, saliency maps were generated for healthy subjects and patients with MS to identify brain regions that were deemed important for the brain age prediction task by the CNN.

**Results:**

Overall, the application of the CNN revealed accelerated brain aging with a larger brain age gap for patients with MS with a mean of 6.98 ± 7.18 years in comparison to healthy test set subjects (0.23 ± 4.64 years). The brain age gap for MS patients was weakly to moderately correlated with age at disease onset (ρ = −0.299, *p* < 0.0001), EDSS score (ρ = 0.206, *p* = 0.004), disease duration (ρ = 0.162, *p* = 0.024), lesion volume (ρ = 0.630, *p* < 0.0001), and brain parenchymal fraction (ρ = −0.718, *p* < 0.0001). The saliency maps indicated significant differences in the lateral ventricle (*p* < 0.0001), insula (*p* < 0.0001), third ventricle (*p* < 0.0001), and fourth ventricle (*p* = 0.0001) in the right hemisphere. In the left hemisphere, the inferior lateral ventricle (*p* < 0.0001) and the third ventricle (*p* < 0.0001) showed significant differences. Furthermore, the Dice similarity coefficient showed the highest overlap of salient regions between the MS patients and the oldest healthy subjects, indicating that neurodegeneration is accelerated in this patient cohort.

**Discussion:**

In conclusion, the results of this study show that the brain age gap is a valuable surrogate biomarker to measure disease progression in patients with multiple sclerosis.

## 1 Introduction

Multiple sclerosis (MS) is a chronic demyelinating disease of the central nervous system. It is characterized by areas of inflammation, demyelination, and axonal loss in the central nervous system. White matter (WM) lesions have been considered a hallmark of MS and are typically visualized and quantified using T2-weighted magnetic resonance imaging (MRI) to monitor disease progression. However, only weak correlations of T2 lesion volume with clinical impairment have been found so far (1). This has stimulated further efforts to identify quantitative imaging measures to better monitor pathological processes and progression of MS with relevance for clinical presentation but also for treatment effect analyses. Within this context, it is increasingly recognized that a more widespread and subtle form of inflammation and degeneration occurs within the normal-appearing white matter but also within the cortex and subcortical gray matter (2, 3). Currently approved drugs have been shown to significantly reduce the frequency of attacks of the relapsing MS forms and number and volume of lesions but have shown limited efficacy preventing transition to the progressive phase and neurodegeneration (4). Thus, the rate and stage of neurodegeneration rather than the lesion load is becoming more important when evaluating new drugs.

The rather subtle inflammation and degeneration in the normal appearing white matter and gray matter can cause significant brain atrophy, which can be observed even in the earliest stages of the disease (5). Cerebral atrophy is a common feature of many neurological diseases and is typically associated with the loss of neurons and their connections (synapses). Atrophy can be localized to specific brain regions or occur as a global process affecting all or most brain areas. It is typically associated with specific or broad and irreversible neurological and cognitive impairments. Whole-brain atrophy has emerged as a clinically relevant biomarker for progression analysis in many diseases, including MS (6, 7). Within this context, multiple studies have shown that brain atrophy is better correlated with neurological and cognitive impairment compared to the lesion number and volume (8, 9). Thus, in addition to reducing inflammation, preventing neurodegeneration has become an important treatment target. Due to this, brain volume measurements are used more frequently in recent randomized clinical trials to evaluate the effect of treatment approaches to reduce atrophy. Global and regional brain volumes can be measured *in vivo*, for example, using high-resolution T1-weighted MRI datasets employing automatic image analysis methods (10). A common approach to segment different structural or functional brain regions is to use an atlas-based approach, in which an atlas with a known brain parcellation is registered non-linearly to a specific subject dataset. After registration, the structural or functional atlas brain regions can be transformed to the patient anatomy. Simple volume quantification derived from cross-sectional data, however, cannot be used to determine the current atrophy stage or rate directly, since atrophy refers to a volumetric change due to cell loss over time.

Moreover, changes in brain volume do not only occur due to MS, as normal aging, lifestyle factors, genetic predisposition, and other pathophysiological diseases also affect brain volumes and lead to atrophy (11). As a result, atrophy trends are seen across one's lifespan, but can show considerable inter-subject variability (12).

The so-called brain age gap (BAG) may be a sensitive, comprehensive, and therefore better alternative to calculating regional brain volumes for obtaining an understanding of the general brain health and cumulative effect of diseases like MS (13). The BAG denotes the difference between the chronological age and the biological age predicted using machine learning methods based on brain imaging data. Typically, machine learning models for brain age prediction are trained using high-resolution T1-weighted MRI datasets directly or tabulated imaging data from a large number of healthy subjects covering the full age spectrum. Within this context, it has already been reported that the biological brain age of patients with MS is considerably older compared to their true chronological age when using a brain age prediction machine learning model based on extreme gradient boosting (14, 15), ordinary least squares regression (16), or Gaussian processes regression (17, 18). However, most previously described studies that investigated the BAG in MS patients only employed traditional machine learning models based on tabulated or numerical data extracted from images. More recently, studies have investigated the effect of MS on brain age prediction using MRI datasets as direct input for a deep learning model based on convolutional neural networks (CNNs) (18–20). However, none of these studies used explainable artificial intelligence (AI) methods to identify which areas of the images actually contribute to the increased BAG and thus may be important targets for the development of new drugs and treatments.

Therefore, this study aimed to predict the brain age gap in an MS population by training an explainable deep learning model on a large multi-cohort database of T1-weighted MRI datasets from healthy subjects. Saliency maps were then generated and used to identify the most important brain regions that contribute to the prediction of the biological brain age for healthy subjects and MS patients. Finally, the Dice coefficient was calculated to compare the saliency maps between different age groups.

## 2 Materials and methods

### 2.1 Datasets

For this research study, cross-sectional T1-weighted MRI scans from healthy subjects were retrieved from different databases acquired in various centers around the world. More precisely, five databases containing a total of 5,324 MRI scans from healthy adults (2,914 females, 2,410 males), aged 19–89 years, were used in this work, including the Study of Health in Pomerania (SHIP) (23), Information eXtraction from Images (IXI),^1^ Southwest University Adult Lifespan Dataset (SALD) (24), Dallas Lifespan Brain Study (DLBS),^2^ and Open Access Series of Imaging Studies (OASIS-3) (25). For these studies, all subjects were classified as healthy based on varying criteria, *e.g*., no neurodegenerative or neurological diseases, no brain pathologies, and normal cognitive function (26). For the MS dataset, MRI scans of 201 patients with relapsing remitting multiple sclerosis (RRMS) (148 females, 53 males), aged 20–60 years were available and used in this work. The inclusion criteria for the healthy subjects and the MS patients can be found in Table 1. The MRI scans and associated demographic data of the MS patients were originally acquired in the Pilot Trial of Domperidone in RRMS (clinicaltrials.gov identifier NCT02493049). Participants also consented to participate in an observational study that permitted use of their images, demographic, and clinical data for this analysis (The Clinical Impact of MS: University of Calgary Ethics ID REB 14-1926). The demographics and scanning details of all included data can be found in Table 2.

**Table 1:** Inclusion criteria of the datasets included in this work.

**Database**	**Inclusion criteria**
SHIP	No known pathologies in T1 scans
	No awareness of the presence of any neurodegenerative disease
IXI	All subjects were healthy without any known disease
SALD	No known psychiatric or neurological disorders
DLBS	All participants underwent cognitive testing and were rated as having normal cognitive function (mini-mental state exam score of 26 or above)
OASIS-3	All participants underwent cognitive testing and had a clinical dementia rating scale score of 0 at the time of imaging
RRMS	Patients recruited in MS Clinic in Alberta, Canada
	Main inclusion criteria: • Confirmed MS according to the McDonald criteria (21) • Confirmed diagnosis of RRMS according to Lublin et al. (22) • On treatment with disease-modifying therapy for at least 6 months

**Table 2:** Demographics and scanning details of the datasets included in this work.

**Database**	**No. of patients**	**Sex (M/F)**	**Age range**	**Mean age + SD**	**Type of scanner**	**Resolution (mm)**
SHIP	3,215	1,555/1,660	21–89	52.55 ± 13.71	Siemens 1.5T	1.0 × 1.0 × 1.0
IXI	560	250/310	20–86	48.62 ± 16.50	Philips 1.5T & 3T GE 1.5T	0.9375 × 0.9375 × 1.2 0.9375 × 0.9375 × 1.2
SALD	494	187/307	19–80	45.18 ± 17.44	Siemens 3T	1.0 × 1.0 × 1.0
DLBS	314	117/197	21–89	54.53 ± 20.05	Philips 3T	1.0 × 1.0 × 1.0
OASIS-3	741	301/440	45–89	68.04 ± 8.90	Siemens 1.5T & 3T	1.0 × 1.0 × 1.25
RRMS	201	53/148	20–60	44.40 ± 8.88	GE 3T	1.0 × 1.0 × 1.0

### 2.2 Data preprocessing

All T1-weighted MRI datasets were preprocessed using the publicly available Advanced Normalization Tools (ANTs) software.^3^ For bias field correction, the N4-bias algorithm was applied in a first step (27). Next, each MRI scan was spatially normalized to the MNI brain atlas using an affine registration, which preserves the specific local brain morphology for each subject (28). Using a binary mask, all datasets were skull-stripped to remove any non-brain tissue from the imaging data, which is not of interest and can negatively affect the subsequent analyses if not removed. For this, the binary mask available in atlas space was non-linearly transformed. This non-linear transformation was calculated for each subject by mapping the MNI brain atlas to each MRI scan. After skull-stripping, the intensities of the brain image were rescaled to a zero mean, to ensure that the intensity range of each image is comparable, even across studies (29). Finally, all MRI scans were cropped as much as possible without removing any brain information. By removing all unnecessary background voxels across the population, the size of the transformed images was reduced from 182 × 218 × 182 voxels to 162 × 198 × 164 voxels, thereby considerably reducing computational costs in the following steps.

After applying all preprocessing steps, the final datasets were visually inspected, and subjects were excluded when skull stripping failed, when a motion artifact was present, or when registration results were not deemed suitable.

### 2.3 Prediction of biological brain age

**Figure 1:**
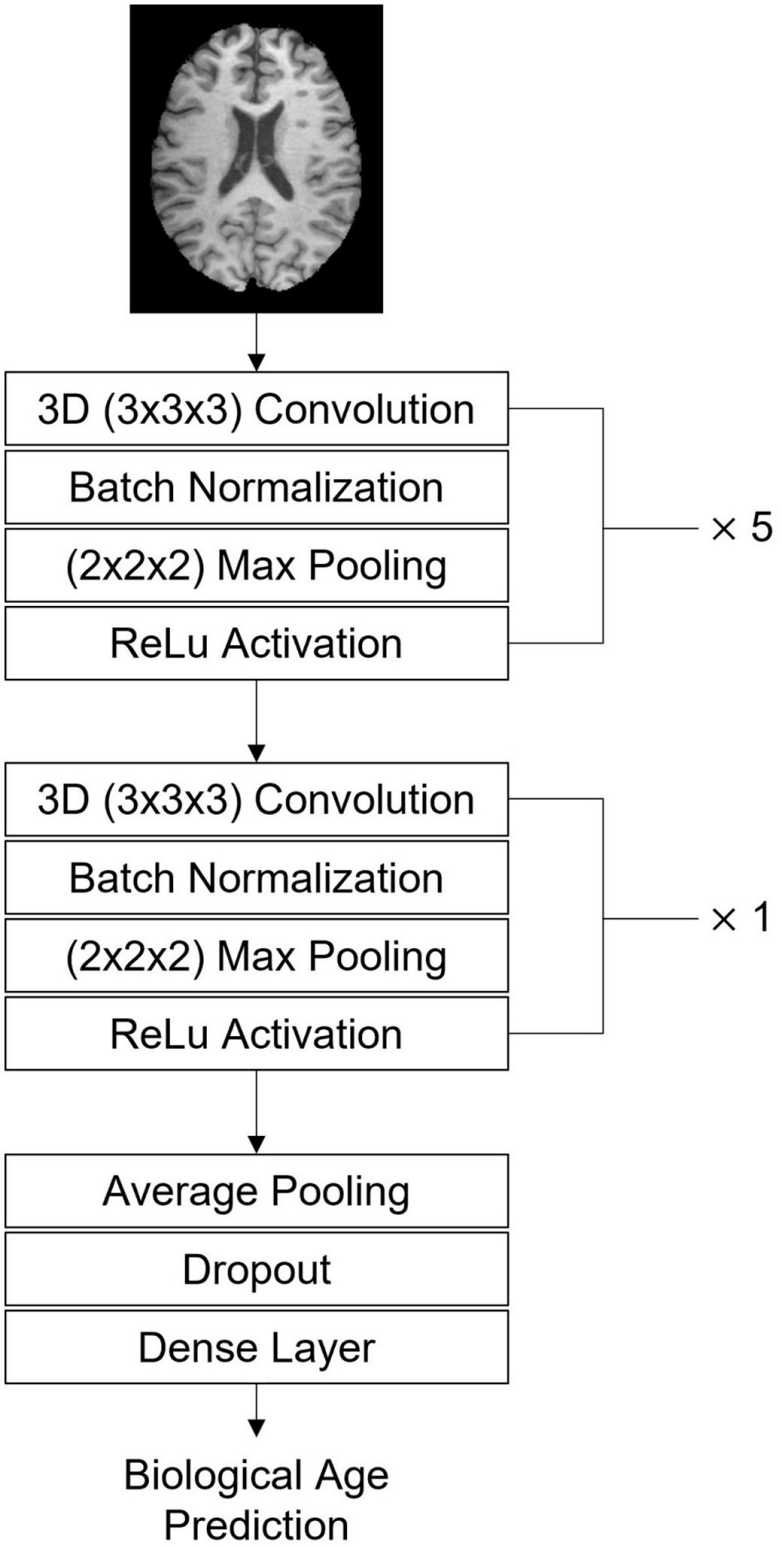
The architecture of the CNN for predicting brain age using 3-dimensional T1-weighted MRI scans as input and returning the biological brain age in years as output.

The prediction of the biological brain age was performed by training a CNN model, with a healthy training set as input, for whom we assumed that the biological brain age is equal to the chronological age. The model used for this purpose in this work is based on the Simple Fully Convolutional Network (SFCN) described by Peng et al. and the CNN architecture presented in Mouches et al. (29, 30). The complete model used in this work consists of seven blocks (Figure 1). The first five blocks all contain a 3-dimensional 3 × 3 × 3 convolutional layer, a batch normalization layer, a 2 × 2 × 2 max pooling layer, and ReLu activation function (30, 31). These blocks contain 32, 64, 128, 256, 256, and 64 filters. After that, a block was added containing a 3-dimensional 1 × 1 × 1 convolutional layer, a batch normalization layer, and a ReLu activation function with 64 filters. The final block of the model contains an average pooling layer, a dropout of 0.5, and a dense layer with a linear activation to return the predicted brain age as the final output value.

All included healthy subjects were split into training, validation, and testing sets in the ratio of 75/22.5/2.5%. The allocation of the subjects to the three sets was conducted with age and sex as stratification criteria, to generate similar distributions of the included data for each set. To reduce the risk of overfitting of the model, data augmentation was ranomly applied to 50% of the training data using ±5 voxel random translations (29). The final model was trained with 300 epochs, making use of the Adam optimizer with a learning rate of 0.001, a weight decay of 0.0003, and a batch size of 8, which was also used by Mouches et al. (29). During the training process, the validation set was used to determine the best mean absolute error (MAE), used for optimizing the weights for the final model. The remaining 2.5% of the data from healthy subjects was used as a small test set to perform an accuracy evaluation by calculating the MAE.

### 2.4 Brain age gap estimation in MS patients

After the CNN model was trained and the optimal weights were found using the data from the healthy subjects, the model was used to predict the biological brain age of all available MS patients (not used at any point for the model development). Next, the difference between the chronological and the biological brain age was computed (*i.e*., brain age gap) for each subject.

### 2.5 Saliency maps

To identify the most predictive regions for this brain age prediction task, the SmoothGrad method was used to create gradient-based saliency maps for the healthy individuals used in the test set as well as for all available patients with MS (32). The SmoothGrad method creates the saliency map by assigning a certain importance to each voxel of an MRI image. Next, each saliency map was transformed non-linearly to the MNI brain atlas and then linearly mapped to an intensity range of [0, 255].

For a quantitative comparison of the final saliency maps between the healthy individuals and MS patients, noise was removed from the saliency maps (33). This was achieved by thresholding out the bottom 5% of voxel intensity values. For each raw saliency map, a weighted saliency score was computed for each brain region as defined in the CerebrA atlas (28). This score was calculated as described by Stanley et al. (33) by computing a saliency score as the percentage of non-zero intensity voxels for each brain region. Since higher intensity values reflect more important regions, the saliency score is corrected by multiplication with a weighting factor. This factor was calculated by computing the average intensity value in each brain region for each saliency map and scaling these mean intensities to a range of [0, 1] using min-max scaling. The higher the saliency score, the more important the region is assumed to be for an individual in predicting brain age.

As a final evaluation, the Dice similarity coefficient was calculated to measure the overlap between the binary saliency map of the healthy subjects and those of the patients with MS for the four age groups. For each of the age groups 30–39, 40–49, and 50–59, the saliency maps of twenty randomly selected subjects (10 males and 10 females) were averaged. Due to the low number of individuals within the age group below 30, only 10 subjects could be included consisting of five males and five females. After each thresholded saliency map was transformed into a binary map, the Dice coefficient was calculated for each comparison.

### 2.6 Statistics

Basic brain volume measurements were computed for each patient with MS for the purpose of correlation analyses. Therefore, the Sequence Adaptive Multimodal SEGmentation (SAMSEG) tool from the FreeSurfer package (V.7.4.1) was used (34). This tool enables the quantification of the overall lesion volume (in ml) as well as the volume of regional brain volumes (in ml). The global white matter volume was calculated by combining the regional cerebral white matter volumes. Likewise, the global cortical gray matter volume was calculated by combining all cortical gray matter volumes, while the global sub-cortical gray matter volume was calculated by combining all corresponding regional deep gray matter brain volumes. These brain volumes were normalized by dividing them by the estimated intracranial volume (eTIV). Finally, the brain parenchymal fraction (BPF) was calculated as the sum of the white matter volume and the total gray matter volume (cortical and subcortical gray matter) and was divided by the eTIV.

Statistical analyses were conducted using R (version 4.3.1). The Mann–Whitney *U*-test was used to determine statistically significant clinical variables that affect the brain age gap in MS patients. Furthermore, Spearman's correlation coefficient (ρ) was calculated to discover any link between clinical variables and global brain volume variables and the BAG in the MS cohort. Additionally, a partial correlation was calculated to determine the degree of association between the BAG as the dependent variable and clinical and global brain volume variables as independent variables while controlling for chronological age and sex with the exception of the age at onset variable only being controlled for sex. Statistically significant differences in salient regions between healthy individuals and MS patients were computed using the Mann–Whitney *U*-test. Because there were 102 brain regions analyzed between the two groups, correction for multiple testing was applied to compensate for rejecting the null hypotheses by chance. This was done using the Bonferroni correction (35). For all tests, statistical significance was considered for α < 0.05.

## 3 Results

### 3.1 Data inclusion

After preprocessing the datasets, a total of 30 healthy subjects were excluded because of deformations or incorrect skull-stripping or registration results. Additionally, six scans from patients with MS had to be removed because of inaccurate skull-stripping or severe motion artifacts. As a result, a final number of 5,294 healthy subjects (2,899 females, 2,395 males) and 195 patients with MS (146 females, 49 males) were included in this work. A detailed overview of the clinical characteristics and global brain volume variables of the MS dataset can be found in Table 3.

**Table 3:** Patient characteristics of multiple sclerosis cohort.

**Variable**	**Median [1st–3rd quartile]**	
Age (years)	45.00 [38.00–51.20]	
Age at onset (years)	31.90 [26.60–38.45]	
EDSS score	2.00 [1.50–3.00]	
Disease duration (years)	10.20 [5.30–17.25]	
Treatment duration (years)	2.20 [1.10–3.30]	
Lesion volume (ml)	1.05 [0.19–3.31]	
Normalized lesion volume (%)	7.80e-04 [1.34e-04–2.25e-03]	
Cerebral white matter volume (ml)	387.96 [360.31–421.82]	
Normalized cerebral white matter volume (%)	0.257 [0.248–0.266]	
Cortical gray matter volume (ml)	462.70 [429.34–497.87]	
Normalized cortical gray matter volume (%)	0.307 [0.299–0.316]	
Subcortical gray matter volume (ml)	51.53 [48.61–55.04]	
Normalized subcortical gray matter volume (%)	0.034 [0.033–0.036]	
Total gray matter volume (ml)	609.77 [571.76–655.75]	
Normalized total gray matter volume (%)	0.406 [0.394–0.420]	
Brain parenchymal fraction	0.68 [0.66–0.70]	
**Disease-modifying therapy (DMT)**	**Name**	**Count**
	Aubagio	7
	Avonex	3
	Betaseron	3
	Copaxone	37
	Gilenya	62
	Minocycline	4
	Plegridy	3
	Rebif	14
	Tecfidera	68

### 3.2 Prediction of biological brain age

Table 4 shows the mean absolute error (MAE) and the BAG of the trained model when tested using data from the held out healthy subject test set and all patients with MS. Overall, the brain age prediction model achieved an MAE of 3.67 years for the healthy subjects, which is within the range of reported values in previous research (26, 29). For the five different studies individually, a comparable MAE range of 3.57–3.90 years was achieved, as shown in Table 5. For the MS patients, the MAE was found to be considerably higher compared to the healthy data (MAE = 7.98 years), indicating that the biological brain age for MS patients differs more from their chronological age than it does for the healthy subjects. More precisely, applying the trained model to patients with MS led to a mean BAG of 6.98 years (SD = 7.18) compared to 0.23 years (SD = 4.64) for healthy subjects.

**Table 4:** Model accuracy for biological brain age prediction of healthy test subjects and MS patients.

**Dataset**	**Mean absolute error (SD)**	**Mean BAG (SD)**
Healthy	3.67 (2.83)	0.23 (4.64)
MS	7.98 (6.03)	6.98 (7.18)

**Table 5:** Model performance for biological brain age prediction of healthy test subjects per database.

**Database**	**No. of subjects**	**Mean absolute error (SD)**
SHIP	160	3.62 (2.71)
IXI	27	3.90 (3.14)
SALD	22	3.57 (3.34)
DLBS	17	3.70 (3.67)
OASIS-3	36	3.80 (2.46)

**Figure 2:**
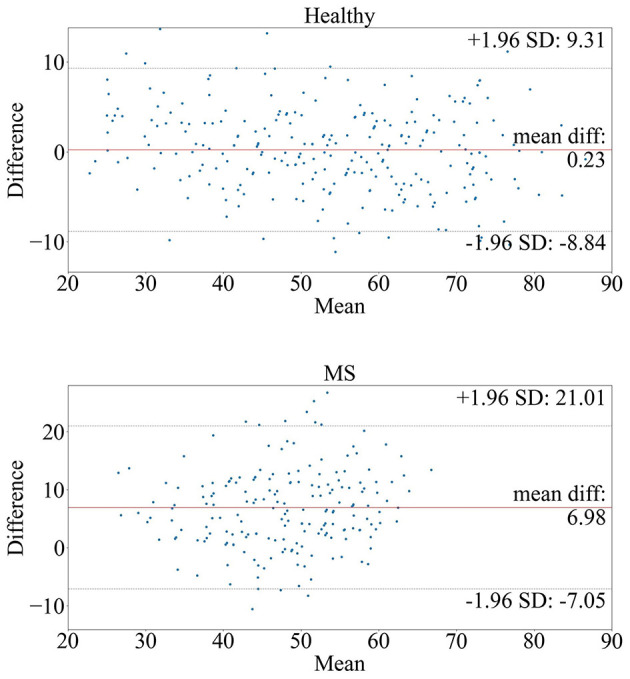
Bland-Altman plots of the model tested on **(top)** healthy subjects and **(bottom)** MS patients. The plots compare the true and predicted age by visualizing the mean true age in years (*x*-axis) and brain age gap in years (*y*-axis) between the true and predicted age.

**Figure 3:**
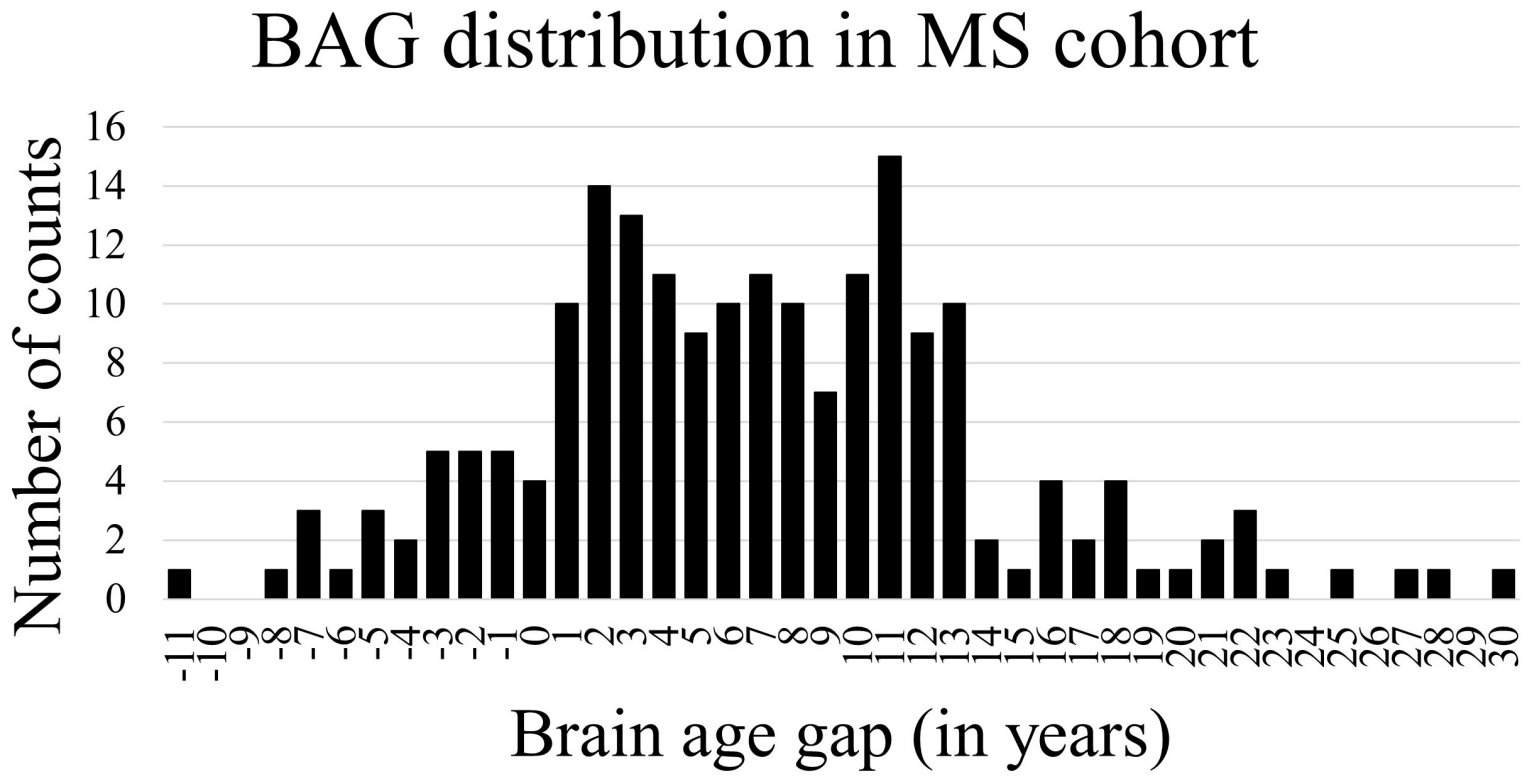
Distribution of brain age gap in years for the MS cohort.

Figure 2 shows the Bland-Altman plots visualizing the performance of the trained deep learning model on the dataset from healthy subjects (Figure 2 top) and MS patients (Figure 2 bottom). The Bland-Altman plot displays the mean of the true chronological and predicted age on the *x*-axis and the actual difference between these two ages on the *y*-axis. The plot for the MS subjects indicates a wider 95% confidence interval of the data with a distribution toward a higher difference (–7.05; 21.01) compared to the healthy subjects (–8.84; 9.31), indicating that the predictions of the brain age for patients with MS were predominantly older than their true age with a larger variability. Figure 3 shows the distribution of the BAG in years for the MS cohort. In total, eleven patients showed a BAG below zero, indicating a younger-appearing brain. Table 6 shows the characteristics of the MS patients with a younger-appearing brain and those from the MS patient cohort with a BAG equal to or above zero. This overview shows that the chronological age, the age at disease onset, and EDSS score are significantly different between these two groups.

**Table 6:** Clinical characteristics of MS patients with a BAG <0 and those of MS patients with a BAG above or equal to zero (≥0) are shown as the median and the interquartile range (IQR), calculated as the third quartile minus the first quartile.

**Variable**	**BAG < 0 median [IQR]**	**BAG ≥0 median [IQR]**	***p*-value**
Chronological brain age	49 [6.5]	43 [14]	0.003
Age at disease onset	37.8 [11.6]	31.3 [11.9]	0.005
EDSS score	1.5 [1]	2 [1.5]	0.006
Disease duration	9.6 [12.2]	10.3 [12]	0.908
Treatment duration	2 [1.6]	2.2 [2.2]	0.473

The rightmost column shows the *p*-value of the Mann–Whitney *U*-test used to calculate statistical differences between the two groups.

The univariate correlation analysis comparing the BAG of the whole MS cohort shows weak to moderate but significant correlations with age at disease onset (ρ = –0.315, *p* < 0.0001), normalized lesion volume (ρ = 0.586, *p* < 0.0001), normalized cerebral WM volume (ρ = –0.512, *p* < 0.0001), normalized cortical GM volume (ρ = –0.436, *p* < 0.0001), normalized subcortical GM volume (ρ = –0.479, *p* < 0.0001), normalized total GM volume (ρ = –0.468, *p* < 0.0001), and BPF (ρ = –0.559, *p* < 0.0001; see Table 7). No statistically significant correlations were found between the BAG and any other clinical variable investigated. The partial correlation analysis revealed a significant correlation between the BAG and the age at disease onset (ρ = –0.299, *p* < 0.0001) when controlled for sex. For the other clinical variables, significant correlations were found for EDSS score (ρ = 0.206, *p* = 0.004) and disease duration (ρ = 0.162, *p* = 0.024) when controlled for chronological age and sex. All global brain volume variables investigated were significantly correlated with BAG when controlled for chronological age and sex; normalized lesion volume (ρ = 0.630, *p* < 0.0001), normalized cerebral WM volume (ρ = –0.516, *p* < 0.0001), normalized cortical GM volume (ρ = –0.647, *p* < 0.0001), normalized subcortical GM volume (ρ = –0.534, *p* < 0.0001), normalized total GM volume (ρ = –0.713, *p* < 0.0001), and BPF (ρ = –0.718, *p* < 0.0001).

**Table 7:** Univariate and partial correlation using Spearman's rank correlation coefficient (ρ) between the brain age gap and clinical characteristics and global brain volume variables for the MS cohort.

	**Univariate**		**Partial**	
**Variable**	ρ	*p* **-value**	ρ	*p* **-value**
Age at disease onset	–0.315	< 0.0001	–0.299^a^	< 0.0001
EDSS score	0.140	0.051	0.206^b^	0.004
Disease duration	0.029	0.691	0.162^b^	0.024
Treatment duration	0.003	0.961	0.011^b^	0.875
Normalized lesion volume	0.586	< 0.0001	0.630^b^	< 0.0001
Normalized cerebral white matter volume	–0.512	< 0.0001	–0.516^b^	< 0.0001
Normalized cortical gray matter volume	–0.436	< 0.0001	–0.647^b^	< 0.0001
Normalized subcortical gray matter volume	–0.479	< 0.0001	–0.534^b^	< 0.0001
Normalized total gray matter volume	–0.468	< 0.0001	–0.713^b^	< 0.0001
Brain parenchymal fraction	–0.559	< 0.0001	–0.718^b^	< 0.0001

^a^Corrected for effect of sex.

^b^Corrected for effect of chronological age and sex.

### 3.3 Saliency maps

**Figure 4:**
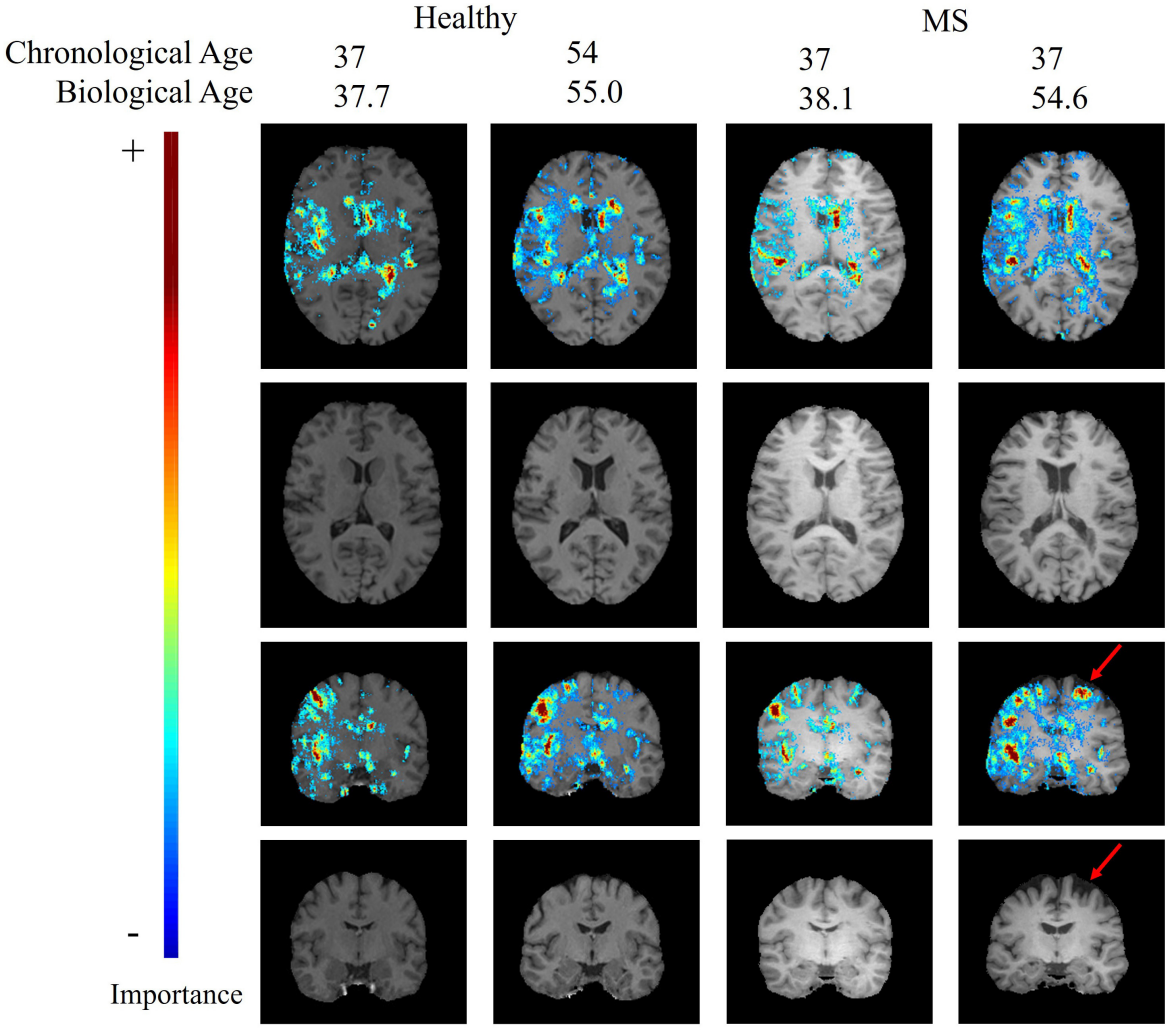
Age-specific T1-weighted MRI with and without saliency maps from an axial and a coronal view of two healthy subjects and two MS patients. The red arrow indicates a patient-specific region with brain atrophy caused by neurodegeneration.

Figure 4 shows an axial and a coronal slice of T1-weighted MRI brain images and corresponding saliency maps of two selected healthy subjects and two selected MS patients used for brain age prediction. The predicted biological brain age is close to the original chronological age for the healthy subjects and for the MS patient with a predicted age of 38.1. The MS patient shown on the right has a chronological age of 37 but is predicted to be the same age as the older healthy subject with an age of 54. The saliency maps indicate that the trained model focuses on the brain regions corresponding to the ventricles, particularly the lateral ventricles. The gyri are also deemed important by the CNN model for each subject, with the region around the right temporal gyrus showing high saliency values. There are also several sulci that are highlighted in the saliency maps.

**Figure 5:**
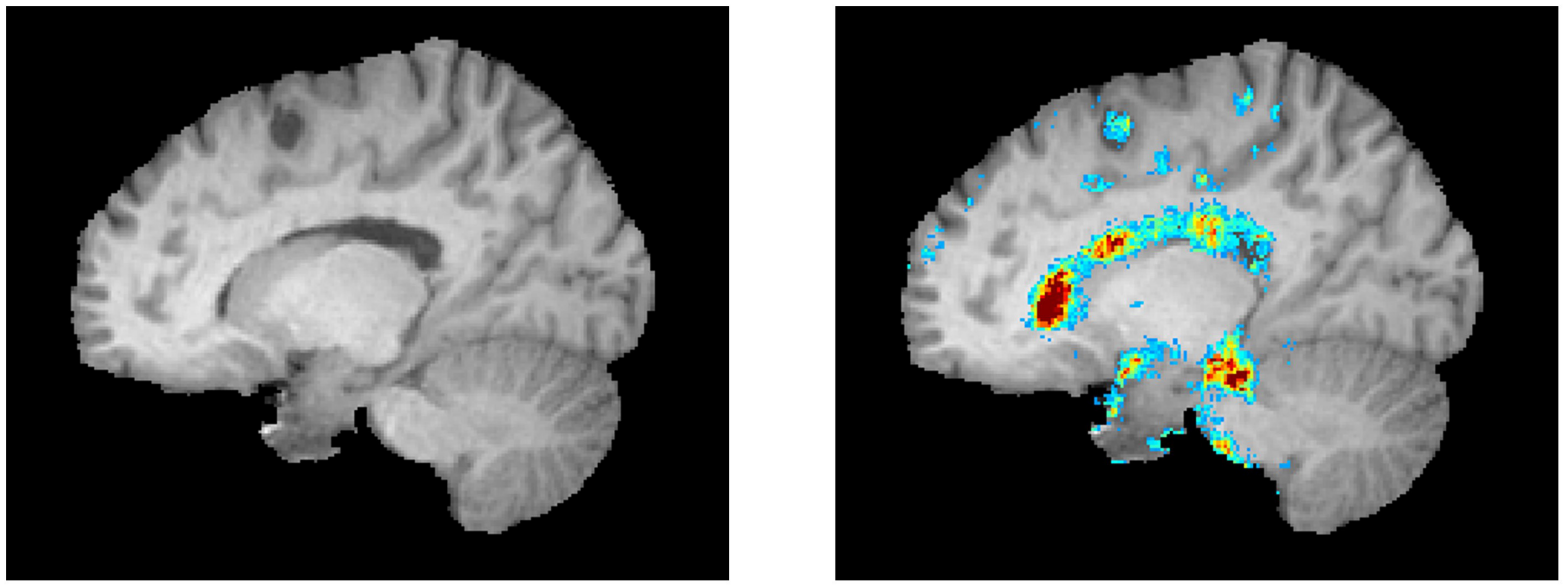
A lesion is visible as a black hole on the left. The corresponding saliency map is shown in the right image (same color scale as Figure 4).

The top regions in these maps are defined by the highest weighted saliency scores. Comparing the saliency map of the MS patient, who is estimated to be 17 years older than their true age, with the other saliency maps, it becomes apparent that the saliency map is more comparable to the 54-year-old healthy subject. This similarity is also observed in the size of the ventricles, as seen in the T1-weighted MRI slices. In addition, a region that is not found in the other saliency maps but is present in the age-overestimated patient with MS is the region indicated by the red arrow, corresponding to a region of the brain that is highly affected by neurodegeneration. Figure 5 shows a sagittal slice of the MS patient that is predicted to be almost as old as their true age. A black hole is visible on this slice, which corresponds to a chronic lesion. Although this lesion is highlighted in the saliency map, the importance is rather small and has no large effect on the prediction of the model as its biological brain age is close to its chronological age.

**Figure 6:**
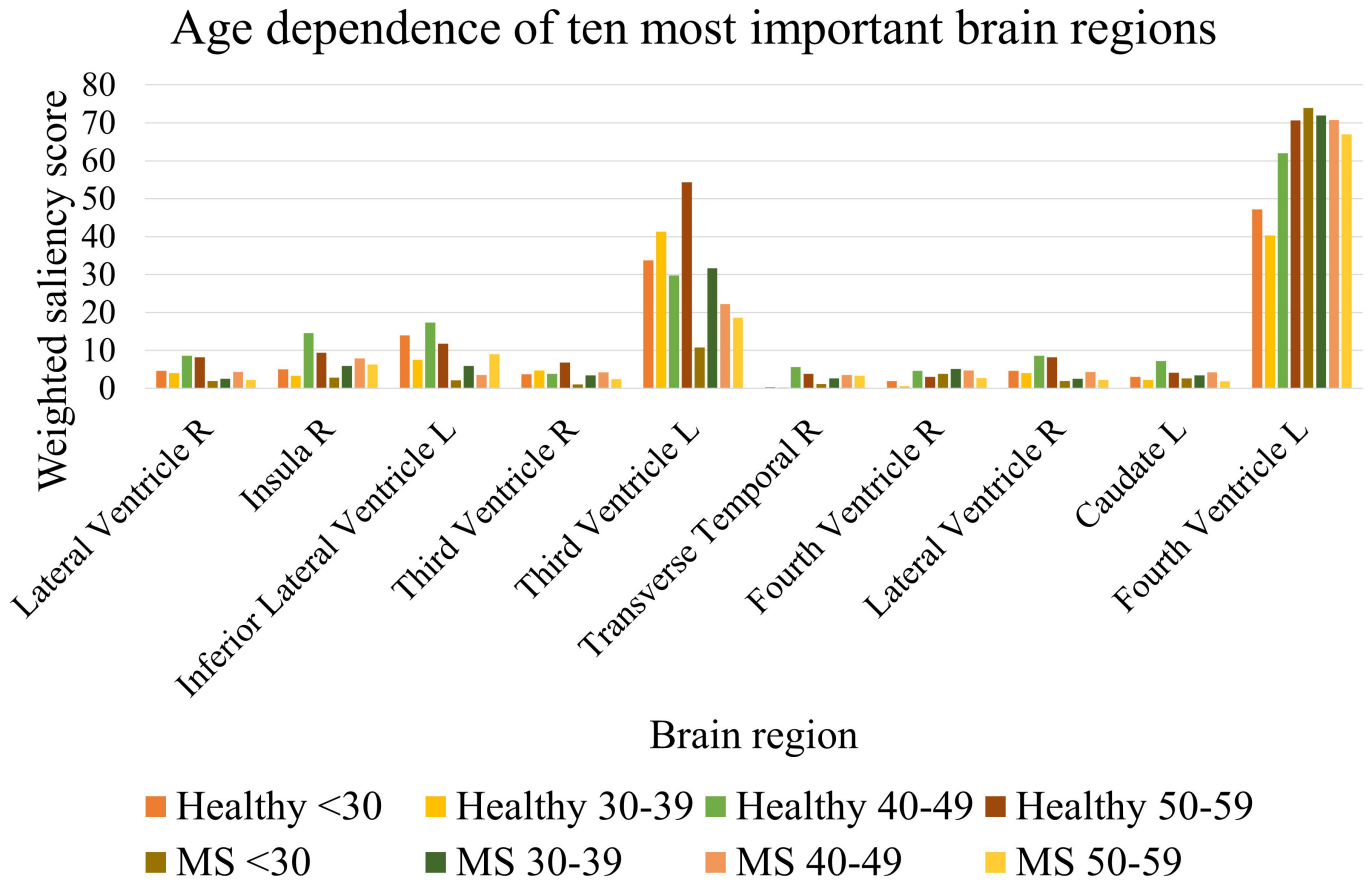
Age dependence of the 10 most important brain regions for brain age prediction for each age subgroup of healthy subjects and MS patients.

The Mann–Whitney *U*-test was used to compare the weighted saliency scores for each brain region between all healthy subjects and MS patients between the chronological ages of twenty and sixty. To account for multiple testing, a finding was considered significantly different with α = 0.05/102 (*p* < 0.00049). The 10 most important brain regions for the prediction model, according to the average magnitude of the saliency scores of the healthy testing group, are presented in Table 8. A complete overview of the significance values of all brain regions can be found in Supplementary Table 1. Table 8 shows that the most important regions for the biological brain age prediction task are significantly different between the healthy group and MS group, except for the left lateral ventricle (*p* = 0.001), left caudate (*p* = 0.002), and the left side of the fourth ventricle (*p* = 0.136). The most significantly different region found is the lateral ventricle in the right hemisphere. Figure 6 shows the age dependence of these 10 brain regions for both healthy subjects and MS patients, subdivided into four age groups (< 30, 30–40, 40–50, 50–60). This analysis demonstrated higher scores for the majority of these regions for the older age subgroups of both healthy and MS, with a lower overall score for the MS subgroups, except from the fourth ventricle.

**Table 8:** *p*-values of Mann–Whitney *U*-test with multiple testing correction for saliency map comparison of the 10 most important regions for brain age prediction.

**Region**	**Bonferroni corrected *p*-value (*p* < 0.00049)**
Lateral ventricle R	**< 0.0001**
Insula R	**< 0.0001**
Inferior lateral ventricle L	**< 0.0001**
Third ventricle R	**< 0.0001**
Third ventricle L	**< 0.0001**
Transverse temporal R	**< 0.0001**
Fourth ventricle R	**0.00014**
Lateral ventricle L	0.00112
Caudate L	0.00201
Fourth ventricle L	0.13566

A *p*-value < 0.00049 indicates statistical significance (in bold).

**Figure 7:**
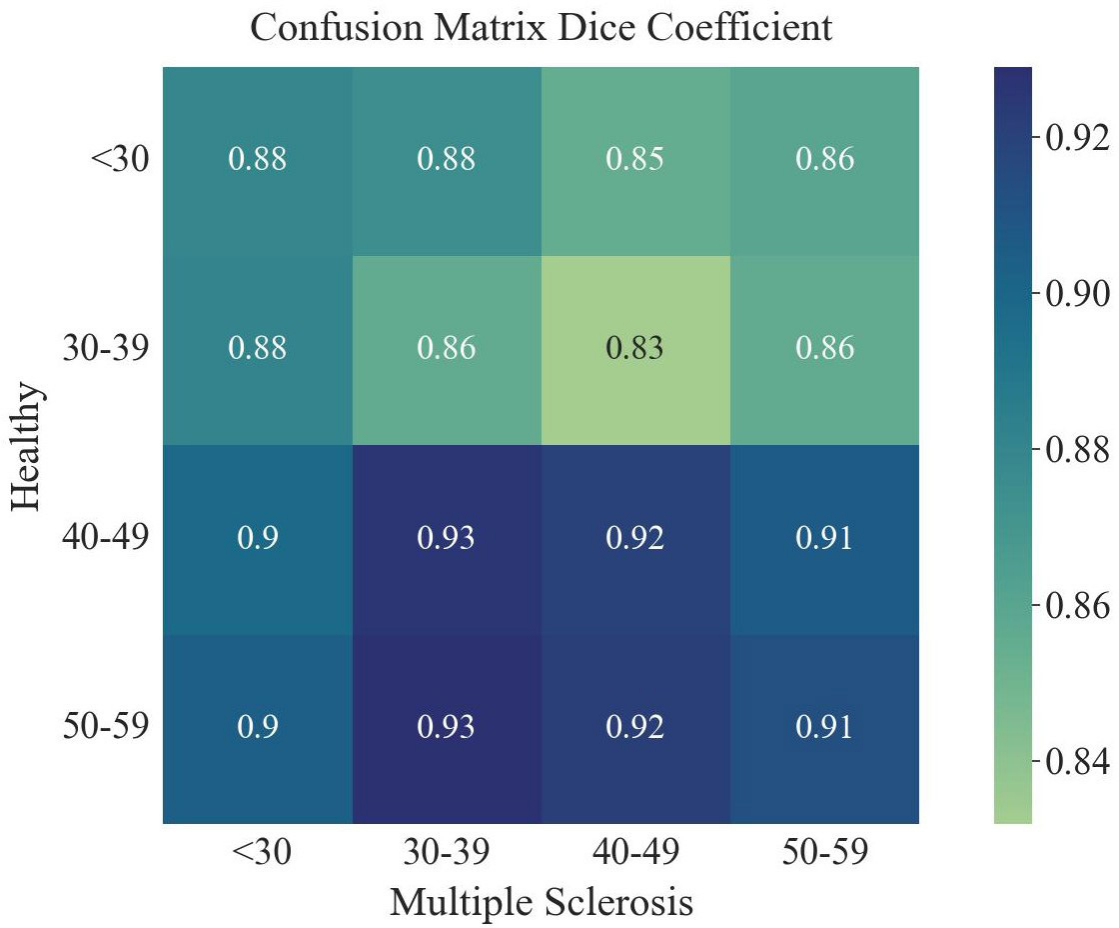
Dice coefficient for the overlap of each averaged saliency map of the age groups of healthy and MS.

The overlap of a saliency map from healthy subjects and one from the MS patients can be calculated by the Dice similarity metric, in which a value of one reflects perfect overlap. This coefficient was determined by comparing the averaged and binarized saliency map of each healthy subgroup and MS subgroup and is plotted in Figure 7. Overall, the Dice similarity coefficients range from 0.83 to 0.93, with the lowest overlap found between the saliency maps of the younger healthy subgroups and the older subgroups of MS. The highest Dice coefficient, reflecting the largest overlap, was found between the 40–49 and 50–59 age groups of healthy subjects and the 30–39 age group of patients with MS. Calculation of the overlap between an MS subgroup and an older healthy subgroup resulted in a higher Dice coefficient compared to the overlap with the healthy subgroup of the same age range. The oldest two healthy subgroups led to the highest Dice coefficients for all MS subgroups.

## 4 Discussion

In this study, a deep learning model was trained to predict the biological brain age based on structural T1-weighted brain MRI datasets and applied to patients with MS to gain insights into the structural differences of the brain in this patient cohort compared to healthy individuals using explainable AI methods. Training the model on a large number of healthy subjects and testing the trained network on both healthy subjects and MS patients revealed a higher variability between the true and predicted brain age in MS patients than in healthy subjects. The brain age gap was only weakly to moderately correlated with other clinical or global volumetric brain measurements. In addition, the most predictive brain regions responsible for individual brain age predictions were identified by saliency maps. An additional comparison was made by calculating the overlap of the average saliency maps between different age groups. By carrying out these steps on both the patients with MS and the healthy subjects, clinically feasible differences between the two groups were found to exist.

The average brain age gap of 6.98 ± 7.18 years found for the MS patients is considerably higher than the brain age gap found for healthy subjects (0.23 ± 4.64 years). These findings suggest that our proposed brain age prediction model could be used as a biomarker for revealing accelerated brain aging in the group of MS patients and is in agreement with previous deep learning research investigating the biological brain age in MS (18–20). For example, Schulz et al. (19) computed an MAE of 6.18 ± 4.83 when applying the DeepBrainNet model for brain age prediction in a cohort of MS patients. The predicted BAG was 10.08 ± 8.99 for MS patients and 3.95 ± 6.82 for healthy subjects (19). The CNN model used in that study was pre-trained on 11,729 healthy subjects and used the median age of all predictions across each MRI-slice as the predicted brain age, which is a significant difference from the CNN model developed in this work, which uses the full 3D spatial information (19). Moreover, their dataset also included secondary-progressive MS patients, which may contribute to the increased brain age gap as this group was also predicted older in a study by Cole et al. (36). Within the study of Wei et al. (20), a BAG of 13.09 ± 14.7 was found for the MS subjects when using a SFCN with a BAG of 0.8 ± 6.2 for the healthy controls. A potential reason for this discrepancy can be the use of a more advanced patient cohort (20). However, none of the previous studies used explainable AI methods to investigate what brain regions differ between the patient cohort and healthy subjects and may be new treatment targets of interest.

The comparison between the MS patients with a younger-appearing brain (BAG < 0) and all other MS patients showed a statistical difference for some clinical variables. It was found that the group with a BAG below zero was older (*p* = 0.003) and developed MS at a later age (*p* = 0.005). Furthermore, this group also had a slightly better EDSS (*p* = 0.006). The associations between a younger-appearing brain and the age at onset and EDSS were also reported by Brier et al. (18). However, when computing the correlation coefficient between the BAG and clinical variables in the whole MS cohort, the EDSS score was not significantly correlated with BAG (ρ = 0.140, *p* = 0.051). In contrast, Wei et al. (20) did find a significant correlation between EDSS worsening and BAG, but with a median EDSS score of 3.5 [3] for their RRMS group, which is higher than the EDSS score of the MS cohort used in our study (2.00 [1.50]). Nevertheless, the association became significant (ρ = 0.206, *p* = 0.004) when the partial correlation between EDSS score and BAG was calculated with correction for the effect of chronological age and sex. Moreover, the partial correlation did show a weak, but significant, correlation between the age at disease onset and the BAG (ρ = –0.299, *p* < 0.0001) when corrected for sex and between the disease duration and the BAG (ρ = 0.162, *p* = 0.024) when corrected for chronological age and sex. This is in line with a study of Cole et al. (36) that described a relation between higher BAG values and a higher EDSS score, a longer disease duration, and a younger age at diagnosis. Furthermore, the partial correlation also revealed significant but only moderate correlations for the normalized lesion volume (ρ = 0.630, *p* < 0.0001), cerebral WM volume (ρ = –0.516, *p* < 0.0001), cortical GM volume (ρ = –0.647, *p* < 0.0001), subcortical GM volume (ρ = –0.534, *p* < 0.0001), total GM volume (ρ = –0.713, *p* < 0.0001), and brain parenchymal fraction (ρ = –0.718, *p* < 0.0001). While this indicates moderate correlations between the brain age gap and an increase in lesion volume and a decrease in BPF, which is related to brain atrophy, it also suggests that the brain age gap is a composite score that takes into account various MS pathologies, including the lesion load and global and local atrophy patterns. Overall, these results suggest that the BAG is indeed suitable as a disease biomarker beyond simple brain volumes and lesion loads.

The saliency maps for healthy and MS subjects revealed that the model trained on a healthy aging baseline focuses especially on brain regions affected by atrophy. This is supported by findings of the Dice coefficient showing that the saliency maps of the oldest groups of healthy subjects, who experienced more normal brain atrophy, had the biggest overlap with the saliency maps of the MS subjects (12). Even the MS subjects with the youngest age range had a bigger overlap with older healthy subjects compared to healthy subjects in the same age range. When investigating the most important regions used for the prediction of the biological brain age, it was found that these were located around the lateral ventricles and the third and fourth ventricle. Of these regions, the third ventricle showed a significant difference in saliency score between the test subjects and the patient cohort, as did the lateral ventricle in the right hemisphere and the inferior lateral ventricle in the left hemisphere. Other important regions that were significantly different between the groups were the right insula and the right transverse temporal gyri. The importance of the ventricles and the involvement of the right temporal lobe in healthy aging is in line with the findings of Scahill et al. (37). The focus on the ventricles of MS patients as well is in agreement with the finding of significant atrophy around the ventricles in RRMS patients (38). Another study found a positive increase in lateral and third ventricle size (39). Interestingly, Simon et al. showed greater enlargement of these ventricles during longitudinal assessment when patients entered the study with enhancing lesions (39). The fourth ventricle was also found to be an important region for healthy brain aging, but did not appear to differ significantly between the MS patients and the healthy cohort. One explanation for this finding might be the inclusion of only RRMS patients, whereas the right fourth ventricle was previously found to be a region of significant atrophy in patients with secondary-progressive MS (38). Nonetheless, the fourth ventricle was more important for each age group in the MS cohort, with higher saliency scores compared to the corresponding healthy age group. The saliency maps in our study also indicate a significant difference in importance of the right insula between the healthy and MS subjects, which is a region that was also indicated in previous studies, where the left insula showed significant atrophy in MS patients (38). Nevertheless, our study did find statistical differences for both, the left and right insula (see Supplementary Table 1), but with the left insula being less important for the brain age prediction task. Although the saliency maps showed similar foci of the model in comparison with other studies and revealed regions with significant importance scores for MS patients, it is not possible to make a statement about the exact causes that result in a bigger brain age gap for these MS patients. Therefore, a future task is to identify in which way the differences between the brain regions affect the predictions of healthy subjects and MS patients in more detail. With this, brain age predictions may be explainable for each individual.

Overall, the biological brain age prediction model that was trained and evaluated in this study achieved an MAE of 3.73 years. Previously reported MAEs range from 2.9 to 5 years for healthy brain age prediction using conventional machine learning methods (20). Although our MAE value is within the range of conventional methods, it is not as low as some of the more recent deep learning-based models. However, there are considerable differences in the data used to test and train those networks as well as a few notable technical differences. For example, the data used in Peng et al. (30) and Wei et al. (20) had a much narrower age range and used a single imaging protocol. Interestingly, our current study used a more robust dataset that included multiple databases compared to a study of Mouches et al. that used only the SHIP database as input, which was also used in this work. Of all SHIP data, only a sample size of 2,074 was used in that study because subjects had to have both good quality T1-weighted and time-of-flight angiography images. This subset of subjects resulted in a slightly worse MAE of 4.01 years (29). When using the same selection of databases and a similar algorithm, an MAE of 3.79 years was found by Wilms et al. (26). Since they used a different pre-processing pipeline, which included the HD-BET algorithm for skull-stripping (40) and the SRI24 template for image registration (41), it may be argued that the pre-processing pipeline does not have a significant effect on the final accuracy of the model. Further limitations that should be acknowledged were the use of five different databases to train the brain age prediction model, which all assumed to include neurologically healthy subjects, although different criteria were used for the definition. Also, the five databases acquired the data using different MRI scanners, which can affect the prediction and may lead to bias (42). However, our study demonstrated a comparable mean absolute error for each database, and previous research has found high reliability between scanners. Therefore, the use of multiple scanners can increase generalizability and make this approach more robust (36). Next, the majority of the healthy subjects were part of the SHIP database, which may result in a bias in the brain age prediction model. Moreover, the prediction of brain age can also be affected by other health issues and lifestyles, like smoking, blood pressure, and the use of alcohol (43). As these factors may contribute to the outcome of the baseline of the model but also to the individual predictions of both healthy subjects and MS patients, a correction could be added to the prediction of the BAG in future research. Additionally, the BAG was not corrected for possible over- and underestimations that can occur for the most extreme age ranges (16). Furthermore, it may be interesting to investigate how highly elevated BAGs differ in any parameter that could have a protective effect. Another issue that needs to be addressed is the rather small sample size of our MS cohort (*n* = 195). Nevertheless, our sample size is comparable to the study of Wei et al. (20) (*n* = 200) who used an SFCN as well. Additionally, it needs to be emphasized that the MS cohort was not used during the training of the model, so that the BAG could be calculated for all MS patients in this cohort using the same model trained on healthy data only. Although our findings are in line with previous literature, the algorithm proposed in this paper should be validated in other cohorts to verify the findings of both the BAG and the saliency maps. Lastly, this study did not directly distinguish between sexes. After pre-processing, the final ratio of healthy males and females in the control group was 45.3% vs. 54.7%, compared to 26.4% males and 73.6% females for the MS cohort. Although the MS cohort has an imbalanced sex ratio, training of the model was only done using healthy subjects with data splitting for training/validation/testing based on age and sex as stratification criteria. However, when analyzing differences between the healthy subjects and the MS patients, a solution is to age-match and sex-match the healthy test group with the MS patients, as was done by Wei et al. (20). No further analyses have been done to compare differences between the outcomes in men and women, but as the imbalanced ratio does not affect the behavior of the model, sex and age were used as confounders when performing partial correlation analyses of the brain age gap. Furthermore, as it was found in other studies that brain volumes differ between men and women, with the ventricles having a larger volume in men, it may be interesting to compare the saliency maps between the sexes (37).

To be able to make additional contributions to the field of personalized medicine (44), it is inevitable to further extend this research as it was limited by the use of only single time point T1-weighted MRI scans. Thus, to improve the performance of this model, it is necessary to include longitudinal data to train the model to differentiate between patient-specific brain changes. This is important as the clinical course can vary significantly among individual patients (4). In addition to inter-patient variability, longitudinal data can be used to train the model to recognize ubiquitous differences between the clinical course of specific brain regions.

In conclusion, this study revealed that a deep learning model trained for biological brain age prediction predicts an accelerated brain aging in MS patients. More precisely, it was found that the lateral ventricles and the third ventricle, and the insula and gyri in the right hemisphere specifically are highly important for the brain age prediction task with a significant difference between MS and healthy subjects, which is in line with current clinical knowledge. The saliency maps showed that the model focused on the regions affected by neurodegeneration resulting in a larger similarity between MS patients and the healthy subjects who are older. Also, the predicted brain age gap was weakly associated with a younger age at disease onset, EDSS score and disease duration, but moderately correlated with a higher lesion volume and lower brain parenchymal fraction. Overall, the results of this work show that the proposed model was able to learn meaningful features from the images that improve our knowledge about specific regions affected by MS that may be potential targets for drug therapy.

## Data Availability

The data analyzed in this study is subject to the following licenses/restrictions. Due to patient privacy, the data used in this work cannot be publicly shared, but can be made available upon reasonable request Requests to access these datasets should be directed to Luanne Metz, lmetz@ucalgary.ca.

## Footnotes

^1^ https://brain-development.org/ixi-dataset/

^2^ http://fcon_1000.projects.nitrc.org/indi/retro/dlbs.html

^3^ http://stnava.github.io/ANTs/
