# Supplementary material for: Analysis and visualization of the effect of multiple sclerosis on biological brain age
Source: Front Neurol. 2024 Oct 10;15:1423485. doi: 10.3389/fneur.2024.1423485 (PMC11499186; doi:10.3389/fneur.2024.1423485)
Supplement: Supplementary file 1 [file Data_Sheet_1.PDF]

SUPPLEMENTARY MATERIAL

Table 1 P-values of Mann-Whitney U-test with multiple testing correction for saliency map comparison for brain age prediction for each brain region as defined in the CerebrA atlas (1). A p-value <0.00049 indicates statistical significance (in bold).

Brain region	P-value (statistical significance of p<0.00049 in bold)
Hippocampus R	**0.0000000000000229**
Inferior Lateral Ventricle R	**0.0000000000011073**
Lingual R	**0.0000000000014009**
Putamen R	**0.0000000000014217**
Lateral Ventricle R	**0.0000000002058903**
Caudate R	**0.0000000017010010**
Thalamus R	**0.0000000081785430**
Inferior Parietal L	**0.0000000251412600**
Amygdala R	**0.0000001445486000**
Insula R	**0.0000006303596000**
Inferior Lateral Ventricle L	**0.0000007860624000**
Fusiform R	**0.0000010466890000**
Pars Opercularis L	**0.0000013099780000**
Pallidum L	**0.0000013421510000**
Insula L	**0.0000013683800000**
Supramarginal L	**0.0000014219590000**
Third Ventricle R	**0.0000026029340000**
Putamen L	**0.0000038888440000**
Isthmus Cingulate R	**0.0000042887480000**
Entorhinal R	**0.0000044838550000**
Entorhinal L	**0.0000071362790000**
Transverse Temporal L	**0.0000074149240000**
Medial Orbitofrontal R	**0.0000082913850000**
Fusiform L	**0.0000116564800000**
Inferior Parietal R	**0.0000314255600000**
Third Ventricle L	**0.0000349761300000**
Brainstem L	**0.0000709768300000**
Transverse Temporal R	**0.0000773167800000**
Fourth Ventricle R	**0.0001414876000000**
Pars Orbitalis L	**0.0001444126000000**
Pericalcarine R	**0.0001598345000000**
Hippocampus L	**0.0001660588000000**
Parahippocampal R	**0.0001853636000000**
Cerebellum Gray Matter L	**0.0001914770000000**
Pallidum R	**0.0002487733000000**
Inferior temporal R	**0.0003847657000000**

Continued on next page

Table 1 – continued from previous page

Brain region	P-value (statistical significance of p<0.00049 in bold)
Caudal Middle Frontal R	**0.0004370672000000**
Inferior temporal L	**0.0004791803000000**
Thalamus L	0.0006783126000000
Pericalcarine L	0.0007144193000000
Isthmus Cingulate L	0.0007172867000000
Vermal lobules VI-VII L	0.0007936901000000
Cerebellum White Matter L	0.0009591944000000
Brainstem R	0.0009732548000000
Lateral Occipital L	0.0011085760000000
Lateral Ventricle L	0.0011207020000000
Vermal lobules VIII-X L	0.0011827180000000
Superior Temporal L	0.0013591020000000
Superior Parietal L	0.0016841730000000
Accumbens Area L	0.0019468470000000
Caudate L	0.0020093050000000
Cerebellum White Matter R	0.0020581730000000
Ventral Diencephalon L	0.0021591980000000
Lateral Orbitofrontal L	0.0030036540000000
Precentral R	0.0030036750000000
Caudal Middle Frontal L	0.0030539290000000
Amygdala L	0.0034502990000000
Precuneus R	0.0050871120000000
Pars Triangularis L	0.0051794750000000
Ventral Diencephalon R	0.0054270310000000
Vermal lobules VI-VII R	0.0087062620000000
Accumbens Area R	0.0089261270000000
Cuneus R	0.0124595200000000
Supramarginal R	0.0129377600000000
Middle Temporal R	0.0130121700000000
Superior Parietal R	0.0174902000000000
Rostral Middle Frontal L	0.0194688800000000
Paracentral L	0.0201164100000000
Vermal lobules VIII-X R	0.0202810500000000
Optic Chiasm R	0.0209515700000000
Superior Temporal R	0.0264164800000000
Lingual L	0.0303951200000000
Pars Opercularis R	0.0304734100000000
Vermal lobules I-V R	0.0421869200000000
Rostral Anterior Cingulate R	0.0485689500000000
Cuneus L	0.0519111700000000

Continued on next page

Table 1 – continued from previous page

**Brain region**	**P-value (statistical significance of p<0.00049 in bold)**
Cerebellum Gray Matter R	0.0779591000000000
Paracentral R	0.0847336000000000
Pars Orbitalis R	0.1129643000000000
Posterior Cingulate R	0.1142455000000000
Pars Triangularis R	0.1169621000000000
Fourth Ventricle L	0.1356565000000000
Vermal lobules I-V L	0.1527669000000000
Optic Chiasm L	0.1648362000000000
Medial Orbitofrontal L	0.1727354000000000
Postcentral R	0.1900686000000000
Lateral Occipital R	0.1977662000000000
Caudal Anterior Cingulate R	0.1991998000000000
Lateral Orbitofrontal R	0.2348872000000000
Superior Frontal R	0.2564698000000000
Superior Frontal L	0.2713048000000000
Postcentral L	0.2832676000000000
Parahippocampal L	0.2979460000000000
Precentral L	0.3148414000000000
Basal Forebrain R	0.3157078000000000
Precuneus L	0.3298148000000000
Rostral Middle Frontal R	0.4429410000000000
Basal Forebrain L	0.5948472000000000
Rostral Anterior Cingulate L	0.6632382000000000
Posterior Cingulate L	0.8940094000000000
Caudal Anterior Cingulate L	0.9336873000000000
Middle Temporal L	0.9584738000000000

REFERENCES

1 .Manera AL, Dadar M, Fonov V, Collins DL. Cerebra, registration and manual label correction of mindboggle-101 atlas for MNI-ICBM152 template. *Scientific Data* **7** (2020) 237. doi:10.1038/s41597-020-0557-9.
